# Synergy between Acceptance of Violence and Sexist Attitudes as a Dating Violence Risk Factor

**DOI:** 10.3390/ijerph17145209

**Published:** 2020-07-19

**Authors:** Inmaculada Fernández-Antelo, Isabel Cuadrado-Gordillo, Guadalupe Martín-Mora Parra

**Affiliations:** Department of Psychology and Anthropology, University of Extremadura, 06071 Badajoz, Spain; cuadrado@unex.es (I.C.-C.); guadammp@gmail.com (M.-M.P.)

**Keywords:** dating violence, sexist attitudes, adolescents, normalization violence, risk factors

## Abstract

The normalization of aggressive behavior in teenage couples when they are dating is a phenomenon that is currently reaching very worrying proportions. The consequences are creating a serious public health problem and have hence aroused the interest of many researchers as to its causes. Most have centered on the role of the aggressor. However, the processes of aggression and victimization are inseparable, and relegating the victims to the background only contributes to increasing the prevalence, severity, and perdurability of the problem. The objectives of this study were to: (i) identify the types and frequency of abuse that adolescents suffer in their relationships; (ii) analyze the relationship between sexist attitudes, acceptance of violence, and victimization; and (iii) determine predictors of the violence suffered in adolescent dating relationships. The sample comprised 2577 adolescents (55.2% girls) of 14 to 18 years in age (M = 15.9, SD = 1.2). The instruments used were the dating violence questionnaire (Cuestionario de Violencia de Novios, CUVINO) and the Scale of detection of sexism in adolescents (Escala de Detección de Sexismo en Adolescentes, DSA). The results indicate that victims showed high tolerance towards gender violence. Acceptance was greater the more frequent the abuse or aggressions suffered. Regarding sexist attitudes, only those belonging to the benevolent dimension had predictive value. The results also show that the interaction between acceptance of the abuse suffered and the manifestation of benevolent sexist attitudes predicted victimization involving specific forms of aggression.

## 1. Introduction

Violence in teenage couples when they are dating is a complex and multi-causal phenomenon. It is generating wide concern in society because of the negative consequences it has on adolescents’ psychosocial development, on a distorted self-perception of healthy romantic relationships between boys and girls, and on their beginning and maintaining future romantic and intimate relationships when they become adults. There are various factors for the normalization of adolescent aggressive and abusive behavior, and for the problems of public health caused by the remarkable increase in cases of teenage dating violence. The absence of physical contact in the new forms of interaction on virtual social networks causes some adolescents to adopt forms of behavior that they would not initiate in their face-to-face exchanges [1,2]. Access to erotic and pornographic videos, without any type of screening, leads some adolescents to take these as referents for the maintenance of their first sexual relations [3,4]. In both girls and boys, copying patriarchal systems and patterns of behavior as well as sexist and discriminatory attitudes towards women [5] is worrying, as also is the false empowerment of women [6]. The perdurability and strengthening of these risk factors causes the victims, mostly girls, to experience a long history of poly-victimization and re-victimization [7].

The difficulties in detecting and identifying situations of violence persist. One reason lies in the physical and social space where they occur—the intimacy of the couple, in many cases not exhibited in public spaces. Another is the degree of acceptance and approval that the victims take on. In this sense, victimized adolescents would feel incapable of describing their relationships as being abusive and justify behavior such as insults, surveillance, humiliation, or emotional deprivation as signs of love [8], fidelity, or characteristic of the male gender [9]. These may even become desirable forms of behavior and relationship [2]. The discrepancies between actual abuse and the perception the couple has of the form of relating to each other has aroused great interest among researchers [10,11,12]. Acceptance of violence is related to processes of aggression and victimization between the members of the couple [13]. In these relationships, boys show greater acceptance of abuse that is committed within a couple [14], making them more likely to be aggressors [7].

Regarding the factors that foster acceptance of violence, some studies point to continued exposure to inter-parental abuse occurring in the family context [14] or to having suffered child abuse [15]. However, not all children exposed to these violent situations become abusers or victims in their dating relationships [16]. There are a number of mediating factors that can help or hinder the adoption of the roles of aggressor or victim. They include moral adjustment [17], adaptive or maladaptive mental schemes [18], the influence of the peer group [19], among others. Likewise, Orpinas et al. [13] warn that the degree of acceptance of violence is linked to the degree of involvement as victims or aggressors, and Karlsson et al. [20] indicate that the level of acceptance of violence decreases as adolescents get older.

Another possible mediating factor between the violence exerted and suffered within the couple and both parties’ acceptance of this abuse, whether aggressor or victim, is the apprehension of cultural and family patterns related to patriarchy and dominance [21]. There have been few studies, however, that have analyzed these relationships in depth, and even fewer that have focused on the indicators predictive of victimization rather than of aggression. In this sense, despite legal, educational, economic, and social initiatives aimed at gender equality, there still remains an evident power imbalance between the roles played by men and women [22]. The forms of discrimination are becoming less explicit because of the social condemnation they face. However, more subtle forms of inequality are emerging that stereotype both men and women, characterizing men as strong, independent, and dominant individuals [23], and women as dependent, sensitive, passive, emotional, and understanding individuals [24]. This macho culture perception is present not only in boys, but also in girls who with all naturalness assume discriminatory gender roles [25]. According to the theory of ambivalent sexism [26], sexist attitudes include a stereotypical view of women that encompasses two perspectives: hostile and benevolent. Hostile sexism reflects a negative affective vision of women and is related to behavioral patterns of dominance and submission, while benevolent sexism acquires a more positive and protective affective tone. One of the most extreme manifestations of sexism is gender violence. In adolescence, this is related to the violence that is exerted within couples [27]. The assumption of these gender roles, together with myths about romantic love that are typical at the adolescent stage, contributes to a search for irrational justifications of the violence exerted and suffered. The reason for this is to minimize the consequences of the experience, and therefore to increase the acceptance of violence as a normalized pattern for relationships, and to make gender violence invisible [28,29].

Moreover, the coexistence of presential and virtual scenarios in which adolescents interact has not only led to gender roles being transferred to cyberspace, but, because of the uncontrolled spread of messages over these platforms and the new reference models to which adolescents look (influencers, youtubers, bloggers, etc.), discriminatory gender differences are being reinforced and certain forms of aggression in couple relationships normalized [30]. Regarding the modalities of aggression, both in offline and online contexts, they have traditionally been classified into three main blocks: verbal-emotional, physical and sexual [31]. Other authors have opted for more detailed classifications that allow the detection of specific types of aggression [32]. In this sense, behaviors such as coercion, humiliation, detachment or emotional punishment that would be part of the same (emotional) category, are analyzed separately to detect the prevalence of some over others. Similarly, in addition to the physical and sexual modality, other specific ones that are directly related to gender violence and instrumental violence are included. For example, in the online context, insults and humiliations on social networks towards the partner tend to be justified as manifestations of jealousy, surveillance through continuous phone calls or by installing geolocation programs are interpreted as signs of protection and love, supervision of the cell phone or hacking social network or email passwords to access and read messages are understood as checking on the degree of loyalty towards the partner, etc. [33]. Such controlling and dominating behavior, fundamentally exercised by boys towards their partners, reinforces conventional roles and causes the continual exposure to this type of action which contribute to the normalization of new forms of violence and the reinforcement of the belief that gender violence has positive effects for the partner [34,35].

During the last decade, studies focused on the search for explanatory and predictive indicators of violence in teenager dating relationships have multiplied [19,36,37,38]. However, most have focused on the description and analysis of the role of aggressor, with the victims being once again the great forgotten [5,21,39]. One of these explanatory indicators is adolescents’ degree of acceptance of violence, although this indicator is generally linked to exposure to previous abusive situations within the family context or to a history of abuse suffered [14,15]. Sexist attitudes have also been associated with the phenomenon of dating violence, fundamentally in a descriptive way [40,41], or with other factors such as alcohol consumption [42]. The interest of the present study is that the role of protagonist is given to the victim, and that its analysis of the factors which contribute to sustaining the role of victim is multidimensional and interrelated. The specific objectives of the study were to: (i) identify the types and frequency of abuse that adolescents suffer in their dating relationships; (ii) analyze the relationship between sexist attitudes, acceptance of violence, and victimization; and (iii) determine predictors of victimization of violence suffered in adolescent dating relationships.

## 2. Materials and Methods

### 2.1. Participants

In this cross-sectional study they have participated a total of 2577 adolescents (55.2% girls) between the ages of 14 and 18 (M = 15.9, SD = 1.2). The selection of this age range is for the moment in which the first relationships of couples begin and, therefore, the moment of early detection of situations of dating violence. These participants were selected following a stratified multistage, approximately proportional, sampling procedure with conglomerates and random selection of groups in public secondary schools in which compulsory secondary education (ESO) is taught. The strata considered were the provinces and geographical areas of Extremadura (Spain), selecting towns in the north, south, east, and west of the region, and taking their different socio-cultural contexts into account. In the urban areas, schools were selected in residential areas in which the purchasing level is medium to high, and in more modest neighborhoods in which people live who normally work in low-skilled trades with a medium to low purchasing power. In the rural areas, the family income was lower than the regional average, and approximately half of the participants’ parents did not have a university education. The conglomerates used were the secondary schools. In each school, one of the four courses (3rd and 4rd secondary school, 1st and 2nd baccalaureate) was selected at random.

### 2.2. Instruments

For the development of this research, two questionnaires designed, validated and used in other research were used:

#### 2.2.1. Dating Violence Questionnaire (Cuestionario de Violencia de Novios, CUVINO)

This questionnaire comprises a total of 61 items [32]. Before these questions, participants are asked for some sociodemographic data such as the family income level grouped into three categories (−900€; 900€ < 2500€; +2500€). Other data such as the level of parental study was provided by each school. The adolescents’ responses provide two main categories of information. On the one hand, they report the types and frequency of aggression they have received from their partners. The modalities considered in the questionnaire are of eighttypes: detachment, humiliation, sexual, coercion, physical, gender, emotional punishment, and instrumental punishment. A five-point Likert scale is used for the frequencies: never, sometimes, frequently, usually, almost always. The reliability analysis (Cronbach’s alpha) obtained from the sample of this study gave values ranging from 0.66 to 0.83 for the types envisaged. And on the other, the item responses allow one to determine the degree of acceptance or tolerance that adolescents have taken on or are willing to take on when their partners abuse them. Specifically, the respondents are asked about their level of discomfort when they experience or might experience the aggressive situations described in the different items. It is understood that the higher the level of discomfort, the lower the level of acceptance and tolerance. The types of aggression are grouped into the same eighttypes: detachment, humiliation, sexual abuse, coercion, physical abuse, gender violence, emotional punishment, and instrumental punishment. The measurement scale used is again a five-point Likert type with 1 being ‘a lot’ and 5 ‘not at all’. The reliability for these modalities ranged between 0.71 and 0.84.

#### 2.2.2. Scale of Detection of Sexism in Adolescents (Escala de Detección de Sexismo en Adolescentes, DSA)

This scale assesses the sexist attitudes that adolescents have towards traditional gender traits and roles, considering two sub-scales—hostile sexism and benevolent sexism [43]. The hostile dimension refers to the traits and roles that place women in an inferior position. The benevolent dimension highlights the qualities of women related to raising and caring for the family. The scale used is a six-point Likert type, with 1 being ‘totally disagree’ and 6 ‘totally agree’. The reliability analysis (Cronbach’s alpha) gave a value of 0.89 for the instrument overall, 0.91 for the hostile dimension, and 0.85 for the benign dimension.

### 2.3. Procedure

Prior to the distribution of the questionnaires to the adolescents, both the research objectives and the procedure, instruments and techniques used were checked and approved by the Bioethics and Biosafety Committee of University of Extremadura (Spain) (Ref. 18/2017). The data collection process was carried out during the 2018/2019 school year. The researchers were the ones who went to schools to pass the paper questionnaires. Once inside the classroom, the researcher gave the questionnaires to each participant who had to fill them out individually. There was no compensation for participating, neither for adolescents nor for schools. Before starting with the distribution and administration of the questionnaires to the adolescents, the schools were approached prior to authorization from the Regional Educational Administration to whom the project was presented, and access to the schools during school hours was facilitated. Then, the management teams of these lower and upper secondary schools were invited to participate in the study, describing to them the objectives and purpose of the study and the use of the data. The written invitation was followed by personal telephone calls to coordinate the collection of data covering the different levels. Once authorizations to enter the schools had been obtained, parental approval was requested (as the participants were minors) by means of a document describing the nature of the study and the mechanisms used to guarantee the anonymity and confidentiality of the responses. The letter was accompanied by an authorization form which the parents had to sign and send back to the school if they wanted their children to participate in the study. The results of the investigation were communicated through a report to the government of the region, which is the financing entity.

### 2.4. Analysis

The analyses were divided into threephases. In the first phase, descriptive analyses were carried out to identify the victims, and then a cluster analysis was applied to check the feasibility of grouping some forms of abuse together for there to be sharper differentiation between them, as well as for the performance and compression of a regression analysis. In the second phase, a correlation analysis was performed to reveal any relationships between the different variables under study. And in the last phase, a negative binomial regression analysis was performed for each of the types of aggression resulting from the cluster analysis.

## 3. Results

To identify the victims, it was considered that the frequency would have to be greater than or equal to the third response anchor of the Likert scale used (never, sometimes, frequently, usually, almost always). The prevalence results indicate that the victims were not subject to a single form of abuse (Table 1), and that the aggressions reported by most victims were detachment, emotional abuse, and coercion.

**Figure 1:**
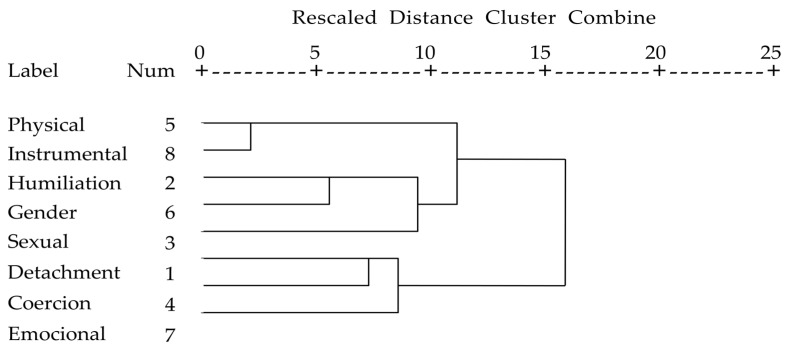
Dendrogram of the grouping of the types of abuse suffered.

The hierarchical cluster analysis allowed the abuse modalities to be grouped together, seeking maximum homogeneity in each group and the greatest differences between groups. The results allowed the modalities to be assigned to three clusters (Figure 1). The first comprised the variables “detachment”, “coercion”, and “emotional punishment” which we grouped under the label “emotional abuse”. The second comprised the variables “physical abuse” and “instrumental punishment” which we grouped under the label “physical abuse”. The third comprised the variables “humiliation”, “sexual abuse”, and “gender violence”, which we grouped under the label “gender violence”.

This clustering of the forms of abuse suffered implies having to group in the same way the forms of aggression towards which the victims show a certain level of tolerance or acceptance. In this way, it can be determined whether there is any type of relationship between the type of aggression suffered and the level of tolerance towards this type of violence. This relationship analysis incorporates the two dimensions of ambivalent sexism (Table 2).

The results indicate that, regardless of the type of abuse they suffered, the victims showed high tolerance towards all kinds of aggression, especially those related to the emotional plane. Acceptance was greater the more frequent the abuse or aggression suffered. Likewise, it was found that the more frequent the abuse suffered and the greater the acceptance of violent behavior, the greater the score in the benevolent sexist dimension but not in the hostile dimension (Table 2).

Finally, the negative binomial regression analysis gave statistically significant models for emotional abuse χ²(4) = 123.78, *p* < 0.001, physical abuse χ²(4) = 78.26, *p* < 0.001, and gender violence χ²(4) = 89.57, *p* < 0.001. The results indicate that the adolescents’ level of acceptance of different types of aggression is a strong predictor of victimization (Table 3). Specifically, high levels of acceptance of emotional abuse were positively associated with the possibility of becoming a victim of emotional abuse (*β* = 0.486, *p* < 0.01), physical abuse (*β* = 0.465, *p* < 0.001), and gender violence (*β* = 0.248, *p* < 0.05). In the same sense, high levels of acceptance of physical abuse predicted the possibility of becoming a victim of emotional abuse (*β* = 0.255, *p* < 0.05), physical abuse (*β* = 0.347, *p* < 0.01), and gender violence (*β* = 0.280, *p* < 0.05). Finally, high levels of acceptance of gender violence predicted victimization of emotional abuse (*β* = 0.364, *p* < 0.01), physical abuse (*β* = 0.408, *p* < 0.01), and gender violence (*β* = 0.377, *p* < 0.01). Also, it was found that boys showed greater acceptance than girls of the abuse suffered or that might potentially be suffered from emotional abuse (*β* = 0.389, *p* < 0.01), physical abuse (*β* = 0.279, *p* < 0.05), or gender violence (*β* = 0.401, *p* < 0.001), making gender a predictor of abuse.

Regarding sexist attitudes, only those that belong to the benevolent dimension have a predictive value of experiencing emotional abuse (*β* = 0.327, *p* < 0.01), physical abuse (*β* = 0.366, *p* < 0.01), or gender violence (*β* = 0.391, *p* < 0.001), with this sexist dimension having higher scores in boys than in girls (Table 3). Hostile sexism is not an indicator of victimization.

The results indicate that the interaction between the acceptance of the abuse suffered and the manifestation of benevolent sexist attitudes predicts the victimization of specific forms of aggression (Table 3). When this interaction occurs on the victimization of emotional abuse, greater acceptance of this type of abuse increases victimization in adolescents with high scores in benevolent sexism (*t* = 4.15, *p* < 0.01). When the interaction between acceptance of physical abuse and benevolent sexism occurs on the victimization of physical abuse, the results indicate that greater acceptance increases the possibility of suffering physical abuse for adolescents with a high level of benevolent sexism (*t* = 3.89, *p* < 0.01). And when the interaction occurs in cases of gender violence victimization, it was found that the greater the acceptance, the greater the victimization in adolescents with high benevolent sexism (*t* = 4.32, *p* < 0.01).

## 4. Discussion

One of the major contributions of this study is the analysis of the level of acceptance of violence by the victims and the impact this has on sustaining their victimization and even converting them into poly-victims. Previous studies have emphasized the figure of the aggressor in trying to understand the motivations that lead them to abuse others, in this case, their dating partner [44,45]. However, the processes of aggression are inextricably linked to those of victimization.

To the extent that those who suffer aggressions rebel and face the situation so as to stop it and prevent future abuse, the incursions of the aggressors will weaken and start to fail. This is not an easy task. The prevalence data show that the victims must confront many difficulties to be able to acquire the necessary tools to combat the scourge of violence. Among these difficulties are those of a personal and cultural nature, which in this study show up in the degree of acceptance of violence and in the manifestation of sexist attitudes.

In relation to the level of acceptance of aggressive behavior in dating relationships, studies such as those by Karlsson et al. [14] suggest that boys are more tolerant than girls in accepting aggressive behavior, partly because they see themselves as the potential aggressors. Our results confirm this tendency of boys being more tolerant of aggression, but it acquired a different meaning. The boys acknowledge that it would not bother them so much that their partners abused them physically, emotionally, socially, sexually, or instrumentally if they thereby maintained their relationship and kept their partner by their side. As Dorado et al. [41] indicate, this could be because boys are less aware of the presence of abusive behavior, and therefore show less rejection of it. This level of acceptance increases the likelihood of boys becoming victims, a topic that has been little studied. However, this role of submission is contradicted when their sexist attitudes are analyzed. The results show that boys score higher than girls in both hostile and benevolent sexism, reflecting a commitment to traditional patriarchal patterns and the exercise of roles linked to domination. These contradictions can be explained by the lack of empathy shown by some boys to put themselves in the victims’ place [46,47]. The interpretation would be that when they admit that they would not be bothered by abuse, in reality what they are revealing is that they do not care that these abuses happen as long as it is they who commit them rather than undergo them. Sánchez et al. [33] explain that boys present some difficulties in recognizing the suffering caused by abusive behavior that takes place in dating, and therefore are more accepting of manifestations of that behavior. The scarcity of studies that have analyzed the processes of victimization to which men are subjected in their couple relationships make it hard to compare results and explore new interpretations.

For girls, the degree of acceptance is lower, but one observes that as the frequency of abuse suffered increases so does the degree of acceptance of the abuse by their partners. Continued submission to aggression leads to its normalization [48]. This is in turn reinforced by the manifestation of benevolent sexist attitudes. García et al. [49] indicate that women recognize the abuse they are subjected to more than men, although, under the umbrella of sexism, they tend to hide their situation of victimization and confer a certain degree of normality on it.

A detailed analysis of the forms of abuse suffered and accepted shows a weak synergistic and mimetic relationship between these variables, demonstrating that the type of aggression suffered is the one with the highest level of acceptance. Thus, victims of emotional abuse tend to show greater acceptance of such abuse than those who have been subjected to physical or sexual abuse. The same relationship is found for the other types of aggression. Exposure to certain forms of aggression may arouse a more active search for justifications that reduce the importance of the suffering experienced, and lead to greater acceptance of the situations being lived through. The need to protect one’s self-esteem so as not to feel victimized, or not to see the increase in pain from the aggressions suffered, stimulates the activation of connection mechanisms of moral disengagement that alleviate this tension and force an acceptance of such abuses [17].

The assumption of sexist roles inherited from tradition, reinforced by the family context and reproduced in the media and by new social agents such as influencers who have become of especial importance for adolescent, places women in a situation of fragility and inferiority [24]. They are oriented towards performing tasks related to the care and protection of children, the home, and the couple [50]. The public role, control, and decision-making are left to men [33]. The present results reflect that 21st century adolescents continue to score high in sexism despite the legal, social, and educational measures adopted to fight for gender equality and opportunities. Although it is true that the hostile dimension is relegated to the background, the benevolent dimension reaches worrying levels, not only in boys, but in girls as well. If boys assume a role of dominance and girls one of submission then girls are accepting a power imbalance in favor of men and implicit patterns of behavior that help them justify and normalize abusive behavior on the part of their partners. All this contributes to the acceptance of gender-related forms of violence and of others related to detachment and emotional punishment, coercion, and humiliation.

The presence of these sexist traits and roles among adolescents has been studied in recent years, confirming that young people continue to reproduce patriarchal attitudinal and behavioral patterns [5,21]. Generally, however, studies of sexism have been associated with the role of the aggressor [51,52] and with the male gender [53]. This present study implies a qualitative leap in understanding the manifestation of sexist attitudes by considering the role of the victim and by including both genders.

Finally, this study’s results also allow progress to be made into the two-way interaction between acceptance of violence and ambivalent sexism, and the repercussions on the victimization of certain types of abuse. In this sense, some previous studies have explored the relationship between the acceptance of violence and sexism [40], although mainly linked to the role of aggressor and in some cases to that of witness [54], associating hostile sexism with the acceptance of dating violence. The present study has found that the relationship between the acceptance of violence and benevolent sexism has a predictive value of victimization. In particular, it anticipates the same type of abuse as that which is tolerated. If the acceptance of violence and sexism alone could be considered risk factors for dating violence, the relationship between the two variables together with the role of victim further defines this risk, and in addition enhances it. It thus constitutes a key element for future programs for the prevention of violence in teenager dating relationships.

## 5. Conclusions

The scourge of gender violence is a global phenomenon detected in all ages and settings, whether presential or virtual. In the case of adolescents, this phenomenon has been widely studied when such violence is between peers (bullying and cyberbullying), but there still remains many questions to resolve when that abuse occurs between the members of a couple who are dating. Knowing the characteristics of the aggressor and the forms of abuse that they use to hurt their partner has been the subject of recent research, and the conclusions have guided numerous intervention measures. Nonetheless, the fight against sexist violence must not ignore the victims. Especial attention should be paid to them and the factors that promote their experiencing abuse and the perdurability of that abuse. This study, which has focused on victims, has provided some keys to understanding the victimization process that occurs in teenager dating relationships (accepting violence and sexist attitudes), keys which can serve to guide new prevention proposals. The role of victim was not only analyzed in women but also in men, describing the most frequent types of abuse suffered. This study therefore provides the identification of risk factors which in many cases may be predictive of dating violence. The results of this research can be considered as a first diagnosis of the situation of violence that takes place in adolescence and highlight the need to work in school on cross-cutting issues such as education in values, gender equality, empathy or respect to others. The main limitation of this study is its transversality. Carrying out longitudinal studies would provide more data on the escalation of violence of these young people when they become adults or on the success of prevention measures implemented in schools.

## Figures and Tables

**Table 1:** Identification of victims of dating violence.

	Boys	Girls	Total
Detachment	110	148	258
Humiliation	38	44	82
Sexual abuse	39	34	73
Coertion	82	93	175
Gender violence	31	59	90
Physical abuse	21	13	34
Emotional abuse	104	84	188
Instrumental punishment	24	18	42

**Table 2:** Correlations between variables.

	1	2	3	4	5	6	7	8
Emotional abuse (EA)	−							
Physical abuse (PA)	0.09	−						
Gender violence (GV)	0.12	0.14	−					
Acceptance emotional abuse	0.24 **	0.19 **	0.22 **	−				
Acceptance physical abuse	0.21 **	0.16 *	0.18 *	0.19 **	−			
Acceptance gender violence	0.17 *	−0.10	0.20 **	0.17*	0.18 *	−		
Hostile sexism	0.07	−0.09	−0.15 *	0.02	−0.03	0.09	−	
Benevolent sexism	0.29 ***	0.27 **	0.19 **	0.20 **	0.20 **	0.22 **	0.15 *	-

* *p* < 0.05; ** *p* < 0.01; *** *p* < 0.001.

**Table 3:** Negative binomial regression analysis.

Predictor Variable	*β*	*p*
**Emotional abuse**		
Gender	0.389	0.003
Acceptance emotional abuse	0.486	0.005
Acceptance physical abuse	0.255	0.048
Acceptance gender violence	0.364	0.009
Hostile sexism	0.79	0.147
Benevolent sexism	0.327	0.007
Acceptance emotional abuse x Benevolent sexism	0.298	0.025
χ^2^	123.78 ***	
*Df*	4	
Physical Abuse		
Gender	0.279	0.024
Acceptance emotional abuse	0.465	0.000
Acceptance physical abuse	0.347	0.008
Acceptance gender violence	0.408	0.002
Hostile sexism	0.114	0.065
Benevolent sexism	0.366	0.004
Acceptance physical abuse x Benevolent sexism	0.261	0.039
χ^2^	78.26 ***	
*Df*	4	
Gender violence		
Gender	0.401	0.001
Acceptance emotional abuse	0.248	0.039
Acceptance physical abuse	0.280	0.028
Acceptance gender violence	0.377	0.009
Hostile sexism	0.046	0.169
Benevolent sexism	0.391	0.000
Acceptance gender violence x Benevolent sexism	0.305	0.014
χ^2^	89.57 ***	
*Df*	4	

*** *p* < 0.001.
